# Immunological Aspects of Isolation and Confinement

**DOI:** 10.3389/fimmu.2021.697435

**Published:** 2021-06-24

**Authors:** Sergey Ponomarev, Sergey Kalinin, Anastasiya Sadova, Marina Rykova, Kseniya Orlova, Brian Crucian

**Affiliations:** ^1^ Laboratory of Immune System Physiology, SSC RF-IBMP RAS, Moscow, Russia; ^2^ Immunology/Virology Laboratory, NASA Johnson Space Center, Environmental Sciences Branch, Houston, TX, United States

**Keywords:** adaptive immunity, space flight, innate immunity, ground-based analogues, immunology of confinement

## Abstract

Beyond all doubts, the exploration of outer space is a strategically important and priority sector of the national economy, scientific and technological development of every and particular country, and of all human civilization in general. A number of stress factors, including a prolonged confinement in a limited hermetically sealed space, influence the human body in space on board the spaceship and during the orbital flight. All these factors predominantly negatively affect various functional systems of the organism, in particular, the astronaut’s immunity. These ground-based experiments allow to elucidate the effect of confinement in a limited space on both the activation of the immunity and the changes of the immune status in dynamics. Also, due to simulation of one or another emergency situation, such an approach allows the estimation of the influence of an additional psychological stress on the immunity, particularly, in the context of the reserve capacity of the immune system. A sealed chamber seems a convenient site for working out the additional techniques for crew members selection, as well as the countermeasures for negative changes in the astronauts’ immune status. In this review we attempted to collect information describing changes in human immunity during isolation experiments with different conditions including short- and long-term experiments in hermetically closed chambers with artificial environment and during Antarctic winter-over.

## Introduction

**Figure 1:**
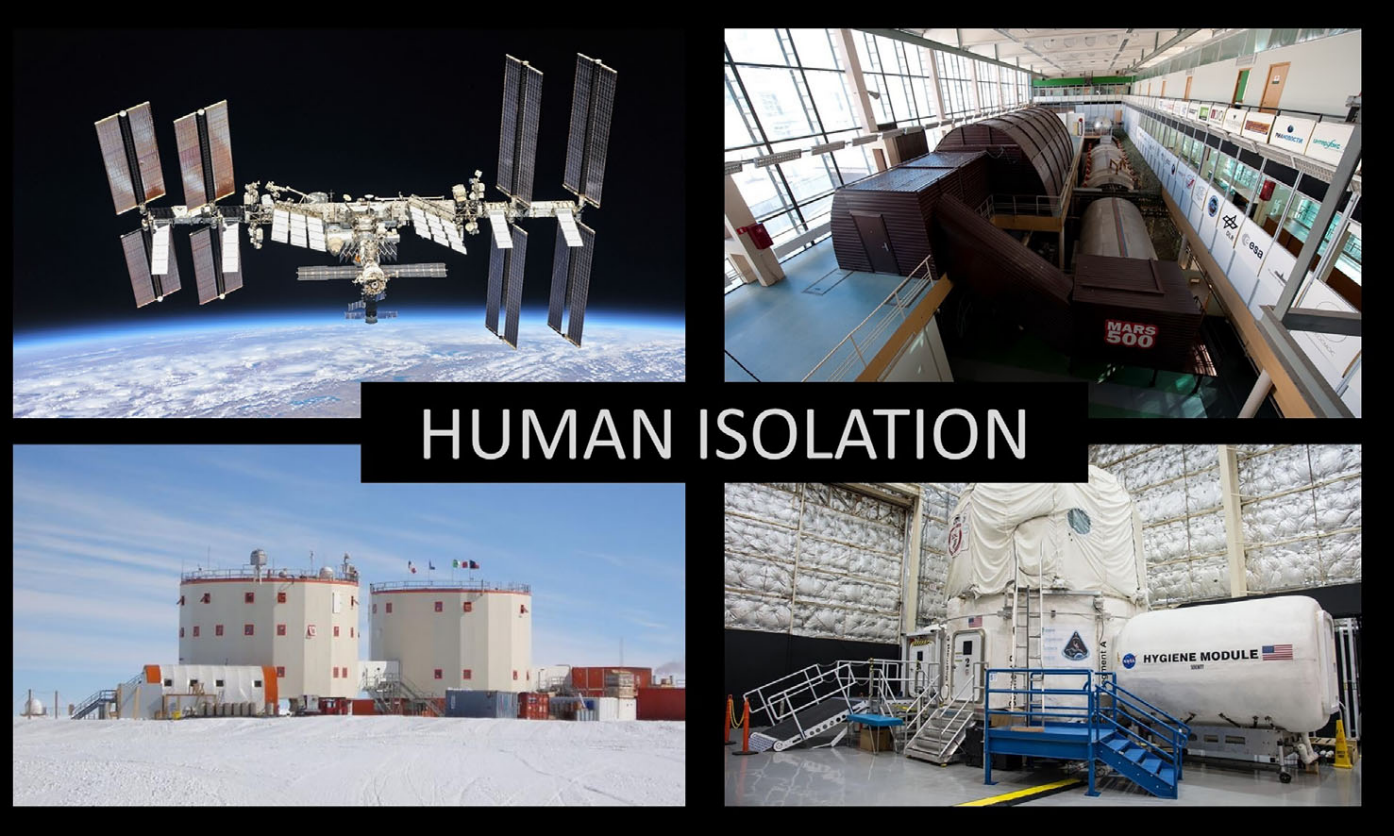
Human isolation facilities. On the left side of the picture displayed the ISS and the Antarctic station (Concordia) . On the right side “terrestrial spacecrafts”: HERA (at the top of the picture) and NEK (at the bottom of the picture).

Immunity is an integral system possessing a quite high level of complexity of its internal organization with a divaricate web of direct and indirect contacts and feedbacks with other physiological systems. The one’s immune status is of special importance in the context of ensuring the normal existence, vital activity and efficiency of a spaceflight crew members. This phenomenon underlies the high individual variability of both the standard values for different indicators and the severity and direction of changes in particular units of the immune system under the influence of the various spaceflight factors. Based on the above, the upcoming missions to the Moon and to Mars require the investigations in the field of confinement experiments of different duration, in which a sealed chamber or Antarctic station simulate the conditions of the environment of a space station, a spaceship, or an on-planet station (**Figure 1**). Each platform has its own variety of factors that could influence on immune system (**Table 1**). During the performance of both short-term and long-term orbital missions the immune system is influenced by a complex of various factors. It is important that, in addition to microgravity, astronauts are subjected to stress and living in a hermetically sealed space with an artificial microclimate. The investigations of immunological reactivity of the crew members of such types of spacecrafts as “Salyut”, “Soyuz”, and “Space Shuttle”, as well as of the orbital stations “SkyLab”, “Salyut 6, 7”, and “MIR”, made it possible to note the changes in the quantity and the functioning of immunocompetent cell subpopulations, and to register the sensibilization of lymphocytes to allergens of bacterial and chemical nature (4–9). Also, during the long-term mission to the station “MIR” a decrease in the delayed hypersensitivity reaction was observed, which indicates a failure in the functioning of cell-mediated immunity (10–12). The analyses of blood, saliva, and urine samples from astronauts revealed multiple well-documented cases of reactivation of such viruses as HHV, EBV, CMV, and *Varicella zoster virus*, in both long-term and short-term missions (13, 14). The researchers noted the shifts in the subpopulation content and the functioning of cells of the adaptive immunity during the stay on board the spacecraft. In particular, a moderate increase in the amount of memory T cells and effector CTLs (CD3^+^CD8^+^ cytotoxic lymphocytes) with a simultaneous decrease in the population of naïve CTLs were observed. In addition, there was an insignificant decrease in the number of cytokine-producing lymphocytes during the flight (15). This study points to the absences of significant changes in the distribution of different lymphocytes subpopulations during the mission. At the same time, considerable shifts in the production of cytokines by the activated immunocompetent cells, as well as in their actual ability to be activated were reported. A failure in the T cells activation upon the contact with staphylococcus enterotoxin and a decrease in the production of IFN-γ, IL-10, IL-6, and TNF-α were observed. In the same manner, the number of lymphocytes capable of specific reaction to viral particles was reduced. However, other studies report an increase in the number of cytokines in the blood plasma of astronauts during a long space mission, with a simultaneous decrease of cytokine production by the mitogen-activated cultured T cells (16). In parallel with these changes, the researchers observed an increase in the number of leukocytes in astronauts.

**Table 1:** Isolation facilities factors.

ISS	Terrestrial Spacecrafts	Antarctic Station (Concordia)
Radiation	Isolation	Hypoxia
Microgravity	Artificial environment	Low temperature
Artificial environment	Psychological stress	Isolation
Stress	Altered nutrition	Artificial environment
Altered nutrition	Hypodynamia (1)	Psychological stress
Hypodynamia		Altered nutrition
Isolation (2)		Hypodinamia (3)

A similar number of changes in the functioning of the immune system are noted in the post-flight period, both immediately after landing and during rehabilitation to terrestrial living conditions. It should be emphasized that in this case the deviations from the pre-flight characteristics described below can be provoked by the prolonged effects of stress during the flight as well as the additional loads caused by the landing process. After a 140-day mission, a decreased proliferative activity of lymphocytes associated with lymphopenia, a decreased cytotoxic activity of NK (natural killers, CD3-CD16^+^CD56^+^) and reduced synthesis of IFN-γ was observed in the crewmembers (6, 17–21). Postflight data obtained during the examination of the crews of the “MIR” station (with the duration of the missions from 66 to 126 days) indicate a decrease in the expression of CD25 by T cells during 48 h incubation with specific activators (14). The studies carried out after a series of short-term and long-term Space Shuttle missions, quantitative and functional changes in the immune system were also noted, namely, an increase in the relative quantity of B-, T-cells (including memory cells), and granulocytes, with a decrease in the relative quantity of monocytes. Additionally, cytokine profile of T-cell response shifted mainly to Th2 response (i.e., IL-10 production predominated over IFN-γ) (22). The results of recent investigations of the ISS crews in the post-flight period also indicate a decreased relative quantity of NK and their reduced cytotoxic activity, weakened functionality of T-cells, and an increase in the total number of the major populations of immunocompetent cells, especially, T-helpers, CTL, CD4^+^CD25^+^-cells, CD4^+^CD45RA^+^-cells, after landing (23, 24). The data obtained during the examination of the ISS crews also confirm the post-flight shift of the cytokine profile of T-cell response towards Th2 response (19). Several researchers reported reactivation of different viruses in the crewmembers both during the mission and in the post-flight period (25–28). Besides, the investigations of innate immunity in the post-flight period indicate a noticeable influence of space flight conditions on it. The inhibition of IL-6 synthesis by monocytes together with the decreased expression of CD62L and HLA-DR on the surface of monocytes were reported after the finishing of a mission, and the production of IL-10, IL-6 and TNF-α by the activated monocytes in culture was especially (by 40-45%) reduced (29). Another study revealed an increase in the absolute quantity of monocytes and granulocytes in astronauts at the first day after landing; the relative quantity of TLR2^+^, TLR4^+^, TLR8^+^, TLR9^+^ monocytes and granulocytes also increased, while the relative quantity of TLR5^+^ and TLR6^+^-granulocytes decreased; the concentration of heat-shock proteins HSP60 and HSP70, which are one of the main ligands for Toll-like receptors, was elevated. These data point to the significant changes in the functioning of innate immunity caused by space flight factors (30). A similar study reported a wide individual variation of both TLR expression on the surface of monocytes and granulocytes and the expression of the corresponding genes, including those coding for proteins from the TLR intracellular signaling pathways, during the first day of the post-flight period. At the same time, the averaged values did not show significant differences between pre- and post-flight values.

The data described above allow us to conclude that the consequences of the impact of the space flight stress factors on immunity are expressed mainly in the reduced reserve capabilities of various immunocompetent cells to response, their phenotypic changes, a quantitative decrease in their effector response through the production of cytokines, and a qualitative shift in the synthesized cytokine profile. Recent findings onboard ISS confirm that the findings delineated above indeed persist during spaceflight, and for the duration of a 6-month orbital mission (31, 32). Particular attention should be drawn to the wide individual variability of changes in immunological parameters which reflects individual susceptibility of the spaceflight factors that should be kept in mind during development of specific immunological countermeasures. Taking into account the fact that future space missions to Mars and to the Moon will require a significant duration and a radically higher degree of crew autonomy, the impact of the flight stress factors on the astronauts’ health will intensify. Since the prolonged isolation in a hermetically sealed space with an artificial microclimate is one of the main stress factors during the mission, Earth-based experiments aimed at a detailed investigation of the stressor on the immunity are of particular importance. An intensive inspection of immunity as a integrate system should be conducted with the regard for the mutual influence of its individual parts on each other. As the immune system is one of the regulatory systems maintaining the organism’s homeostasis, its interactions with other physiological systems (nerve system, endocrine system, etc.), as well as the individual reactions of test-volunteers should be taken into account. The main goal of such investigations should be the development of efficient countermeasures and rehabilitation system for the upcoming space missions, and the detection of precise markers of a current state of the immunity for its correction. The significance of the experiments with the prolonged confinement in a sealed chamber is also conditioned by the global expansion of human labor activity spheres, which is connected to the long-term stay in a closed confined space (e.g., during the development of the sea shelf). Moreover, the data from such investigations may be applied in the elimination of the consequences of the general trend to a reduced physical activity in everyday life and a sedentary lifestyle.

## Experiments With A Confinement

### Experiments With a Confinement in a Specially Designed Sealed Chamber Simulating the Living Space and Closed Environment of a Spacecraft

Spaceflight consists of multiple stressors that synergistically influence humans, including (but not limited to) microgravity, stress, altered nutrition, physiological isolation, and radiation (33). Ground analogs of spaceflight reproduce some of these factors to high fidelity allowing human research to be conducted in a safe, lower cost environment utilizing science capabilities unconstrained as is an actual flight experiment (34). Recent findings suggest that stress is a primary causal factor in the immune dysfunction observed in cosmonauts and astronauts, more so than microgravity or radiation (35). This bodes well for using ground analogs which can closely mimic the isolation and stress of spaceflight to further characterize the problem and develop biomedical countermeasures. While terrestrial ‘deployment’ analogs exist (undersea, Antarctica, etc.), closed chamber confinement in a simulated vehicle for prolonged duration may be particularly useful, as this analog affords the immediate processing of biomedical samples in a fully capable laboratory environment. To conduct the confinement experiments in the conditions of a sealed chamber in the 1960s at the Institute of Biomedical Problems of the USSR Ministry of Health (present IBMP RAS) there was designed and built an Earth-based Scientific-Experimental Complex (the so-called NEK). The NEK, initiated by academician S.P. Korolev, allowed various investigations, including immunological ones, to be carried in the conditions of a pressurized facility with an artificially regulated environment (36). Moreover, the technologies of life support systems, which were then successfully introduced into the manned space missions and flights in orbital stations, had been developed here. Later, the NEK underwent the improvements, among which were the enlargement of a living space, the facilities for testing the equipment and countermeasures development, such as simulators of physical loads and devices for performing physical activities. The crew of IBMP conducted a number of experiments both of short-term (within the frames the mission to the Moon program working out) and long-term confinement in the NEK (within the frames the mission to Mars program working out). Unfortunately, there is no international standard describing conditions of experiments with confinement such as measured parameters, characterizing the state of human immune system, age of test subjects, time points of bio-samples collection, seasons and general experimental conditions including gas composition, pressure, temperature etc. in different isolation projects. This fact makes it difficult to get homogeneous statistics and suggests development of international standard measures in isolation studies.

Terrestrial analogs with closed chamber confinement in a simulated vehicle for prolonged duration may be particularly useful as they allow the immediate processing of biomedical samples in a fully equipped laboratory. In this regard ground analogues could be also used as a platform for the fundamental studies of the interaction between immunity and gut microbiota which seems to play a crucial role in the immune system functioning.

#### Immunity and Microbiome

Microbiome is essential for the immune homeostasis. Immune system has largely evolved as a means to maintain the host’s symbiotic relationship with a variety of microorganisms (37). Dysbiosis usually results in immune reaction such as increased production of different inflammatory cytokines, T cell imbalances, and even autoimmune diseases (38–40). It is well known that space flight associated factors influence human microbiome. The risk of exposure of astronauts and crew members participating in the isolation experiments to the wide variety of pathogenic strains due to the rigorous microbiological control is usually very low therefore a crucial role in dysbiosis belongs to the opportunistic microflora. Changes in bacterial species composition, their gene expression and production of different proteins during space flights and after their completion were observed in a number of space missions and experiments conducted in more than 50 years of the space age (41). Interestingly, similar changes in human microbiota are also observed in terrestrial isolation studies. The stay of a healthy subject in the environment with altered parameters is accompanied by a decrease in colonization resistance of the intestine and integumentary tissues, which leads to dysbiotic changes. Pronounced alterations of the microflora occur in the test-volunteers staying in a hermetically closed chamber with its own microclimate. These are general changes in the intestinal microflora that occur in extreme situations, and the trigger for the dysbiosis development is the decrease in the number of Bifidobacteria and Lactobacilli. The degree of severity of the microflora dysbiotic rearrangement is largely determined by the initial state of the microecological status. In the conditions of a hermetically sealed chamber, the signs of opportunistic microflora activation (represented by Staphylococcus aureus and gram-negative bacteria) were shown together with the quantitative decrease in the normal microflora in the crew members’ microbiocenoses of the mucous membranes of nasal cavity, mouth and throat (42). Till recently, it was not possible to determine the relationship between the changes in the microbiota during the influence of the space flight associated factors and the functioning of the immune system (22). However, the results of the pioneer study provided the first evidence of interaction between human immunity and gastrointestinal microflora during space flights. In this experiment it was shown that the abundance of OTU000010 of the genus Fusicatenibacter negatively correlated with the level of several pro-inflammatory cytokines including IL-1β, TNFα, IL4 and IL-8. In addition, changes in OTU000011 of the genus Dorea also negatively correlated with the changes in the concentration of certain cytokines (namely, IL-1β, IL-1ra, VEGF, and MIP-1b) which were significantly increased during the space missions. Unfortunately, it is impossible to determine yet what was first: the increase in pro-inflammatory cytokines or the decrease in bacteria OTU (43). However, the results of the study are promising and encourage to conduct more experiments to find a greater evidence of the interaction between human immune system and microbiome under the effect of space flight factors. Implementation of different probiotics and other supplements, such as prebiotics or special diets seem to be a viable spaceflight countermeasure (22).

#### Effects of Gender on Adaptation to the Space Flight Factors

One more issue that could be solved in terrestrial analogs is gender dependent immune reaction to the extreme environmental factors. A number of conducted studies reported sex-determined differences in immune responses. Women demonstrate a stronger immune response that manifests in more potent cell-mediated response and enhanced immunoglobulins production (44, 45). This makes women more resistant to microorganisms and viruses and also leads to faster recovery compared to men (46, 47). Men are more prone to different types of hematologic malignancies and solid cancer in non-reproductive organs. The cases of cancer mentioned above significantly more frequent and deadly in males compared to females (48). At the same time women are more susceptible to autoimmune disorders than men. According to Whitacre CC, female gender increases risk of autoimmunity: over 70% of those suffering from autoimmune diseases are women (49). These differences may be explained by the impact of hormones including testosterone, estrogen and progesterone on immune cell functional activity (47). Sex differences in the immune system functioning could be expected during exploration missions. Unfortunately, according to the information available from recent articles based on completed space-flight studies, analyses of sex-determined changes were largely not performed so far (50). Nevertheless, the data obtained during Antarctica winter-over at German Antarctic Research Station Neumayer III with 10 female and 16 male subjects reveled changes in leucocyte subsets and cytokine production, however no significant differences between males and females were observed (51). In another study with 17-days isolation in a sealed chamber no sex-dependent differences in DCs and TLRs^+^ monocytes were shown (52). In our opinion, this phenomenon could be explained by two reasons. The first one and the most likely reason is the lack of statistics and limited analyzed parameters describing the state of immune system. The second one is less likely: there could be no significant differences between men and women in immune reaction to the space flight associated factors but immune response to different antigens could be altered. Both hypotheses need to be validated in experimental conditions.

#### Short-Term Confinement

##### 9-Days Confinement, Female Crew Aged From 25 to 34 Years

The experiment was unique in that the crew of six test-volunteers consisted of women only (53). Other Earth-based simulations had been carried out with the male-volunteers (with rare exceptions), and because of that the issue of the adaptation of female physiology and, in particular, immunity, to the space flight factors remained to be elucidated (54). Additionally, the application of such a unique experimental device as short arm human centrifuge (SAHC) was tested during the 9-day confinement. It is known that the detraining effects of a number of homeostatic systems of the body, including immunity, are observed not only during the space mission itself, but persist for 1.5-2 months upon arrival on Earth (54, 55). In this regard, SAHC seems a perspective and efficient countermeasure for microgravity impact and other stress factors of the flight. Unfortunately, to date, there are no cumulative statistical data on the influence of SACH on different physiological systems of the human body, including the immune system. It is not yet defined when to start training on SAHC as microgravity countermeasure (56). On the one hand, it seems advisable to use the SAHC from the very first days of stay in space in order to ensure smooth adaptation to the conditions of the spacecraft and to prevent the impact of the microgravity adverse effects at the earliest stages. On the other hand, it is possible that the additional load on the human body in the acute period of adaptation caused by rotation on the SAHC will lead to the development of negative changes in many physiological systems of the body, including the immune system. Thus, the study was conducted to define the frames and possible schedule of SAHC application in a real space mission. A special attention was drawn to the first days of confinement as the adaptative period for the new conditions and environment, during which the shifts in the immune system functioning can be observed. In this was it was demonstrated that an intense exchange of microflora between the crew members occurred in the acute adaptative period (during the first 10 days) (57), which, together with the confinement in a sealed chamber, may affect the reorganization in the immune system.

The main factor during the testing of SAHC were the “head-pelvis” overloads (+Gz). The rotation was carried before and after the confinement in the sealed chamber, with the following conditions: acceleration of SAHC to the overload of 1,5 units (at the level of the feet with regard to the Earth gravity) with a plateau of 180s, followed by acceleration of SAHC to the overload of 2 units (at the level of the feet with regard to the Earth gravity) with a plateau of 180s; the total time of rotation was about 14 min. Gravity gradient (Δ G %) was 85% (the head of a test-volunteer was 30cm from the axis of rotation). For immunological studies venous blood was sampled the month before the confinement, after each of the SAHC session, and on the 5^th^ and 9^th^ days of the confinement.

During the investigation a number of changes in the innate and adaptive immunity were revealed. Interestingly, most of the observed changes occur in the innate immunity. To date, one of the fundamental and clinical immunology issues of current importance is the investigation of natural resistance. Innate immune response does not only restrain the expansion of a pathogen till the delayed highly specific adaptive immunity “launch”, but also form a basis for the adaptive immune response development (58, 59). In other words, the innate immune system is the body’s first line of defense against the invasion. The variations of the innate immunity effector mechanisms are defined by specific inherited pattern recognition receptors (PRRs), which may be considered as the carriers of the evolutionary memory of multicellular organisms of how to distinguish between friends and foes (59). The complexity and diversity of these receptors make them efficient in the recognition of a variety of not only exogeneous ligands such as bacterial LPS, fungal mannans, viral nucleic acids, but also endogenous ligands as heat-shock proteins, which result from inflammation and/or tissue damage (60, 61). The changes in such evolutionary conserved signal PRRs as Toll-like receptors (TLRs) play a key role in the early defense of an organism from pathogens, providing the fast recognition of an “invader” and warning of an unapproved invasion of an “alien” which may endanger the organism (61). Upon the interaction of the receptors with their corresponding ligands intracellular signaling pathways are activated leading to the secretion of effector molecules (cytokines, antimicrobial peptides, etc.), to the alterations in the expression of other receptors, and to further adaptive immune response development (60). As a rule, pathogenic and opportunistic microorganisms are considered as “alien” or an “invader”. However, during the recent years it became obvious that TLRs can recognize a number of endogenous agents which evidence a different (non-infectious) danger. Heat-shock proteins (HSP) and uric acid, as well as the products of necrosis and apoptosis are considered such endogenous activators of innate immunity (61).

In the conducted experiment the changes of the subpopulation content, reflecting the percentage (relative quantity) of cells with a particular phenotype in a certain cell pool, and the total number (absolute quantity) of cells expressing one or another surface receptor were observed. The information describing the effects of experimental conditions described above on different cell types is presented below.

**Lymphocytes.** A significant increase in the total number of CD3^+^-lymphocytes but not of their relative quantity in the population of immunocompetent cells was observed during the 5^th^ day of the experiment. The main impact to this increasing was from the T-helpers (CD3^+^CD4^+^). The relative quantity of this very subpopulation of lymphocytes also increased at the 5^th^ day of the experiment. It is worth to note that these changes are in concordance with those observed in the real space flight conditions (22, 23). The application of SAHC before and after confinement in a sealed chamber reduces both the relative and absolute quantity of NK-cells. This may be explained by the immune response to the acute stress. As it is reported in various observations, the shift in the number of natural killers is a typical marker of the immune system stress reaction (53).

**Monocytes and granulocytes.** The changes in the absolute and relative quantity of monocytes expressing the subclass of surface pattern recognition receptors, namely, types 2, 4, and 6 TLRs, were multidirectional, with the same tendency of the dynamic of the changes for all the female test-volunteers in the project. A significant decrease in the number of TLR2+ monocytes was observed after the rotation on SAHC, and the same decrease was noted during the confinement, including the absolute quantity of the cells. The relative number of TLR4+ monocytes increased by the 5^th^ day of the experiment, while by the 9^th^ day of the confinement both absolute and relative quantity of these cells significantly decrease compared to baseline. On the contrary, the number of TLR6+ monocytes decreases dramatically by the 5^th^ day, together with the increasing after the second rotation on SAHC. Dynamic of the granulocytes fluctuations was completely different: after the first rotation there was a tendency to an increase in the absolute and relative quantity of TLR2+, TLR4+, TLR6+ granulocytes. By the 5^th^ day the number of TLR2+ granulocytes decreases significantly compared to baseline, the same was demonstrated for TLR4+ and TLR6+ granulocytes. By the 9^th^ day the population of TLR4+ granulocytes was even more reduced, while the number of TLR6+ granulocytes was significantly increased. The second SAHC session did not affect much the number of TLR2+ and TLR4+ granulocytes, but caused the increase in the absolute quantity of TLR6+ granulocytes. Thus, authors conclude that different cell populations of natural resistance demonstrate heterogenous polymorphism of reactions to the effects of confinement and rotation on SAHC. Authors assume that the differences of such kind result from the functional peculiarities of monocytes and granulocytes, as well as their different sensitivity to the activators. This phenomenon may be also associated with the fact that during the confinement period in a sealed chamber there were a various number of microorganisms (enough for the activation of TLRs) possessing the ligands on their cell walls for different TLRs. The immune system reactions to overloads before and after short-term confinement differ from each other. The changes described above reflect a complex adaptation process happening within the innate and acquired immunity systems during the early period of adaption to the conditions in a hermetically sealed facility and to the rotation on SAHC (53).

##### 17-Days Confinement, Gender-Mixed Crew Aged From 27 to 43 Years

This experiment was conducted to simulate the conditions of the upcoming mission to the Moon with a landing and return to the Earth. Blood was sampled before the experiment from 6 test subjects (3 males, 3 females), on the 7^th^ day of confinement and on the first day of rehabilitation period (52). The experiment was unique in that the impact of confinement on such an important part of immunity as dendritic cells (DCs) was investigated. DCs are the main type of antigen-presenting cells, which means that they play the key role in the transduction of information from innate immunity to adaptive immunity, and in the development of a stable long-term immune response to a particular antigen. Data obtained in the experiment demonstrated changes both in monocytes and DCs.

**Monocytes**. The dramatic decrease in the relative quantity of TLR4+, TLR5+, TLR6+ monocytes, especially, TLR3+, TLR8+, TLR9+ monocytes, by the time of the finishing of the experiment compared to baseline values. The changes observed were gender independent and were noticed in all the crew members.

**DCs.** By the 7^th^ day of confinement an increase in the number of CD14-CD16-CD123+CD85k+ plasmacytoid DCs of the phenotype was observed. Plasmacytoid dendritic cells (pDCs) have lymphoid origin and morphologically resemble plasma cells. They are professional IFN-producing cells that play an important role in the antiviral immune response (62). The revealed shifts indicate that infectious and inflammatory diseases, in particular, caused by the normal microflora of the organism, which does not lead pathological reactions in the host organism under normal conditions, can develop with a short-term exposure to the extreme space flight factors.

#### Long-Term Confinement

The development of a strategy for long-term interplanetary missions, as well as arranging long-term planetary bases, which is a priority goal of space exploration in the long-range outlook, claims a thorough study of the possibility and likelihood of infectious diseases cases, as well as chronic inflammation, allergic reactions and other consequences of immune system disruption in conditions of long-term isolation. In this regard, focusing solely on the extrapolation of the currently accumulated data on immunological changes during orbital missions seems insufficient. The upcoming exploration programs of the Moon, Mars, and other points of outer space imply much longer terms of the missions and much more autonomy. In the light of the above, it became necessary to conduct long-term isolation experiments on the basis of the NEK pressurized facility.

##### 135-Days Confinement, Male Crew Aged From 33 to 38 Years

In this experiment scientists investigated the characteristics of immunological reactivity and allergy status of three test-volunteers who had taken part in the 135-day experiment with the periodical psycho-emotional stress during the stay in a hermetically sealed chamber. The stressful situations simulated during the confinement in the pressurized facility had led to the appearance of abnormalities in the immune system of the test volunteers, including a decrease in the level of A, M, and G immunoglobulins in the blood, a decrease in the functional activity of T-lymphocytes, suppression of the cytotoxic activity of natural killers, and signs of sensitization to bacterial allergens and formaldehyde. The revealed changes are similar to the deviations that are observed in the early period of readaptation to terrestrial conditions after the completion of long space flights (63).

##### 110 and 240-Days Isolation, Gender-Mixed Crew, Aged From 29 to 47 Years

One of the first complex experiments with a comprehensive research of the human immunity was SFINCSS-99 project which consisted of 110-days and 240-days confinements. Three groups of test-volunteers took part in the experiment: the first one consisted of 4 test volunteers who spent 240 days in the pressurized facility, the second and the third groups (of 3 and 4 crew members, correspondingly) were confined for 110 days.

###### Leucocytes

An increase in the absolute quantity of leukocytes by the end of the 110-day confinement, mainly due to an increase of lymphocytes and granulocytes was observed in all three groups (1).

###### Lymphocytes

An increase in absolute quantity by the end of 110-days condiment with no significant differences in lymphocyte subpopulations were observed in all groups (1).

###### T-lymphocytes

The investigation of mitogen-induced blast transformation of lymphocytes in response to PHA in 24h, 48h, and 72h cell cultures in the 4 test-volunteers from the first group demonstrated that the functional activity of T-cells was reduced on average starting from the 4^th^ month of the long-term confinement. Relative quantity of activated T-lymphocytes in a 24h cell culture decreased from 22% to 16%. In a 48h culture, there was a decrease in the activation potential of T cells at the tendency level. In the second and the third groups, a decrease in the activation capacity of T cells was also revealed in certain periods of stay in the hermetically sealed facility. There were individualized dynamics of changes among the crew members of the third group. In the 2 of the test-volunteers the decrease began from the day 39, in the other 2 it started from the 95^th^ day. The decrease on the 95^th^ day was significantly 34% of the baseline values. No significant changes in the T-lymphocytes activation potential were observed in the crew members of the third group. Thus, from the observations described, it is apparent that the functional activity of T-lymphocytes decreased frequently in the conditions of the long-term confinement in the pressurized facility, however, this decrease was moderately pronounced in general and did not go beyond the lower limit of permissible fluctuations of this characteristic in the norm (64). Also, at the end of the 240-day confinement, a significant decrease in the CD4/CD8 ratio was revealed due to a decrease in the proportion of T-helpers (1).

###### B-Lymphocytes, Immunoglobulins

The same research team noted multiple repeated sharp decreases in the immunoglobulins content (in particular, IgM and IgG) at the individual level during certain periods of stay in the pressurized facility, especially in the first three months of the experiment. B-cells count was unaltered. This fact can be regarded as a sign of weakening of the specific function of mature B-lymphocytes clones and lymphoid-plasma cells of the body (secretion of antibacterial and antiviral antibodies) (1, 64). These changes were undulating in nature.

All this taken together indicates a gradual suppression of the adaptive immunity during the period of stay in a pressurized facility, which is expressed in a decrease in its reserve capabilities.

##### 105-Days Confinement, Male Crew, Aged From 25 to 38 Years

Subsequently, based on the data obtained in the SFINCCS experiment, another 105-day confinement experiment was held for a more detailed study of immunological reactions of six male test subjects during long-term isolation in a pressurized facility (65). This study and its results were to form the basis for an experiment on the simulation of the conditions of the upcoming long-term missions to Mars.

###### T-Lymphocytes

The researchers observed an increase in the absolute and relative number of T- lymphocytes at the individual levels during the experiment. The increase in the relative and absolute quantity of lymphocytes in the peripheral blood, apparently, reflects the lymphocytes’ maturation in response to antigenic stimulation, possibly associated with changes in the microbial community of the habitat and the microbial landscape of various biotopes of the human body that arise during the stay in the pressurized facility with artificial environment (66–68). It also indicates an increase in the tension of the adaptive immunity and a shift in the equilibrium of the system towards the intensification of the synthesis of factors that stimulate immune responses. However, on the 7^th^ day after the end of the exposure, there was a clear tendency towards a decrease in the absolute content of lymphocytes in the peripheral blood, which led to a decrease in the absolute content of CD3^+^, CD3^+^CD4^+^, and CD3^+^CD8^+^ cells. On the days 7 and 105, there was an increase in the absolute and relative number of CD4+CD25Bright lymphocytes, suppressing various types of immunocompetent cells that provide both innate and acquired immunity (65, 69). In addition to this, during 2 weeks of the rehabilitation period, the number of CD3^+^CD25^+^ lymphocytes significantly increased, which indicated the activation of the T-cell mediated immunity as a result of prolonged antigenic stimulation *in vivo* (65). The level of CD3^+^CD69^+^ cells in activated cultures steadily decreased by 20% of the baseline starting from the 35th day of the experiment until its completion (65).

###### B-lymphocytes

Analysis of absolute and relative quantity of B-2cells revealed a statistically significant (p<0,05) decrease on the 16^th^ and 70^th^ days of the experiment, as well as on the 14^th^ day after completion of the confinement. Starting from the 16th day of the isolation and until the end of the rehabilitation period, there was an increase in the relative quantity of CD19^+^CD69^+^ lymphocytes in cultures incubated with mitogens (65).

###### Cytokines

The concentration of IL-8 in the blood serum increased in compare to the baseline values during the whole experiment. It should also be emphasized that the analysis of the interactions between the subpopulations of cells involved in the immunological microcompartment and cytokines on the 16^th^, 70^th^, and 105^th^ days of stay in the pressurized facility revealed a significant increase in the total number of reliable connections between them (by 4.2, 1.7 and 2.7 times, respectively; r> ± 0.82; p<0.05), which indicates an increase in the tension of the immune system homeostasis (65). Finally, the results of the studies of the functional reserves of B and T cells in a number of stress tests with mitogens showed that the adaptive restructuring of the human immunity in response to the complex effect of factors of long-term isolation in a pressurized facility required a certain tension of the immune system, as evidenced by an increase in the ability of adaptive immunity cells to be activated and to produce cytokines (65). Authors speculate that such a nature of the shifts indicates that the functional activity of the immunocompetent cell has shifted to a new level corresponding to the conditions of long-term isolation in a pressurized facility.

###### Mononuclear Cells

Another group of researchers also reported an increase in the reactive oxygen species production by mononuclear cells upon TNF-α activation during the stay of the test-volunteers in the pressurized facility, however, the suppression of the phagocytic function of these cells was observed during the same period in a 105-day experiment (70). Activated mononuclear cells showed a much lower level of CD62L expression (the adhesive molecule participating in the phagocyte penetration through the vascular wall during the inflammatory process) during the confinement than during the baseline period. These data indicate a negative effect of isolation conditions on the functioning of innate immune cells, probably due to psychological stress accompanied by the release of stress hormones, which can lead to an insufficient response to an antigen present in a real long-term space mission (70).

##### 520-Days Confinement, Male Crew, Aged From 29 to 40 Years

Finally, in 2010, an experiment with 520-day confinement (Mars-500 project) was carried out on the basis of the NEK facility, simulating a long flight to Mars, circling the planet with landing and staying on its surface, and returning to Earth. In this experiment took part six male test subjects. Within the framework of this study, an extensive array of data was obtained on changes in a wide range of immunological parameters of both innate and adaptive immunity (71).

###### Monocytes and Granulocytes

The relative number of granulocytes decreased during the experiment, although their absolute number remained unchanged (72). Analysis of the data regarding the innate immune system revealed a tendency towards a decrease in the concentration of one of the main endogenous ligands for TLRs, namely, the heat shock protein HSP70, throughout the entire period of isolation, although the differences between the values became significant only by the completion of the experiment. Parallel to this, during the confinement there was a significant decrease in the absolute and relative quantities of monocytes and granulocytes expressing TLR2, TLR4, and TLR6; however, at a number of time points there was a noticeable increase in these parameters at the individual levels. The changes were undulatory by nature, possibly due to the variations in the activation and stagnation of opportunistic microflora. At the individual level, there was a decrease in the number of CD206^+^, and CD16^+^ monocytes and granulocytes on days 248-299 of the experiment. Phagocytic activity of monocytes slightly increased on the 168^th^ day (by 10-15%) with a gradual decrease afterwards. Phagocytic activity of granulocytes increased on the 168^th^, 248^th^, and 360^th^ days of the experiment. Thus, the study of ingesting ability of monocytes and granulocytes, which is one of the effector cellular functions of natural resistance, made it possible to establish that the high activity of phagocytic cells is a characteristic feature of the adaptive restructuring of the innate immunity under the conditions of a long stay in a pressurized facility. The studies of the PRRs made it possible to pioneer the fact that the process of adaptation of the innate immunity to a complex of factors of 520-day confinement in a pressurized object with an artificial habitat is implemented through polymorphic changes in the TLRs of innate immune cells. It should be emphasized that the PRR system is deeply echeloned, and the response to pathogens is provided by the activation of not one receptor, but a certain combination of them, due to which the inactivation of even large PRR groups does not lead to a generalized decrease in the body’s resistance against infections (60, 61). However, the revealed shifts in the PRR system indicate that under the complex influence of extreme factors of the real Martian flight there exist the possibility of the infectious and inflammatory diseases development, in particular, those caused by the normal microflora of the body, which does not cause pathological reactions from the host organism under normal conditions (71).

###### NK-Lymphocytes

The changes caused by the conditions of long-term isolation also affected the natural cytotoxicity system, i.e., the pool of NK cells. After a year of the confinement, the absolute and relative quantities of NKs decreased. In most of the examined test-volunteers, there was mainly a decrease in the content of lymphocytes belonging to the subtype of natural killers with high cytolytic activity (CD56^+^CD16^+^ cells). In two of the six crew members, from the 120^th^ day of stay in the pressurized facility until the end of the observation period, there was a decrease in the relative and absolute quantity of CD56^+^bright NK cells. These results indicate that factors of long-term isolation in the hermetically sealed chamber can cause a decrease not only in the level of circulating NK cells with high cytotoxicity, but also in the level of cytokine-producing NK cells. In addition, a decrease in the expression of the early activation marker CD69^+^ by NKs in response to IL2 activation was noted starting from the 4^th^ month of the experiment. Thus, there is direct evidence of dramatic negative phenotypic and functional changes in the natural cytotoxicity, which, in particular, is responsible for antitumor and antiviral protection. This allows us to conclude that there exist a high probability of both viral reactivation and the risk of pro-oncogenic processes activation in astronauts performing a long space flight. This risk will also increase due to the radiation exposure characteristic to outer space (71).

###### T and B-Lymphocytes

Adaptive immunity is the second (specific) line of immune defense. The advantages of the adaptive immunity over the innate one are 1) higher efficiency, since the accumulated immune factors most fit a specific antigenic stimulus, as well as 2) long, sometimes lifelong, aftereffect associated with the formation of memory cells and maintaining a protective titer of specific antibodies in serum and other biological fluids. The analysis of the data on the changes in the adaptive immunity obtained during the 520-day experiment revealed that the relative and absolute quantities of T and B cells did not, at least, decrease during the entire confinement, but even increased at some time points. At the same time, by the 8^th^ month of the experiment, a significant increase in the proportion of CD4^+^ cells in the population was observed, associated with an increase in the level of both “naive” CD4^+^ T cells (CD4^+^CD45RA^+^) and CD4^+^ memory T cells (CD4^+^CD45RO^+^). In half of the examined test-volunteers, an increase in the relative and/or absolute quantity of CD4^+^CD25^+^Bright lymphocytes was observed during certain periods of stay in the pressurized facility. During the entire period of isolation, the level of produced antibodies of all types did not change, while cytokine response profile of lymphocytes shifted towards Th-2 reactions.

Thus, the use of the functional approach to the assessment of adaptive immunity made it possible to establish that a high degree of tension of the cells of this system is required to maintain homeostasis under the conditions of prolonged exposure to a complex of factors during the confinement in a pressurized facility. The adaptive immune system was mobilized only at the final stage of the experiment, corresponding to the return to Earth. Such a nature of the shifts indicates that the functional activity of an immunocompetent cell transitioned to a new level corresponding to the conditions of long-term isolation in a pressurized facility. But prolonged activation of the immunity in individuals lacking sufficient functional reserves can lead to a depletion of the reserve capabilities of this homeostatic system. Human immune system includes numerous components that have different functions and different degree of specificity to “invading” agents, working as an inextricable balanced whole in a holistic immune system. It is obvious that there exist multiple statistically revealed relationships, both positive and negative ones, between the most diverse parameters characterizing various components of the immune system (73, 74). Therefore, the study of the interaction of these parameters was the basis for the investigation of the immune system from the standpoint of a holistic perception of its functioning during the exposure to the factors of long-term confinement in a pressurized object. Taking into account the strong (up to 2-4-fold) physiological fluctuations affected the absolute quantities ​​of all immunocompetent cells, with the relative number of these cells being more or less stable and changing significantly only in the course of immune reactions, these were the relative quantities of different cell populations and subpopulations in the blood that were taken of all the array of the parameters for the correlation analysis. The results of the study showed that, starting from the fourth month of stay in isolation, there was a decrease in the number of combinations with reliably interrelated parameters (p ≤ 0.05) regardless of the direction of the interactions in the studied fragment, consisting of 528 combinations, including 32 parameters. It should be noted that the decrease in correlations throughout the entire period of the experimental exposure occurred mainly due to a decrease in the number of relationships in the innate immunity, including monocytes and granulocytes, and at the final stage, due to a decrease in the number of relationships between the components of the innate and adaptive parts of the immune system. This phenomenon again testifies for the inextricable interaction between the components of innate and adaptive immunity. At the same time, starting from the sixth month the number of connections in the lymphocytic part of adaptive immunity not only did not decrease, but even slightly increased. Since the magnitude of the integrity of the components reflects the degree of tension of the system, the increase in the interactions can be regarded as the presence of a “tension syndrome” of the adaptive immunity. It seems that the absence of significant shifts in a number of immunological parameters characterizing the content of adaptive immune cells in the peripheral blood during experimental exposure does not yet indicate that this immune system compartment has avoided significant changes. Apparently, these changes are quite intense, but they manifest themselves mainly at a different level, namely, the level of systemic relationships between the components of the immune system (71).

###### Cytokines

An increase in cytokine production by immunocompetent cells was observed upon their activation by the Epstein-Barr viral particles. The concentration of IFNγ and TNF increased, while the concentration of IL-2 did not change. At the same time, there was no increase in the concentration of these cytokines directly in the blood plasma of the test-volunteers compared to baseline. This fact, as well as the absence of a titer of antibodies to this virus and its DNA in blood and saliva, indicates the absence of the phenomenon of viral reactivation in this experiment (72).

In addition, throughout the entire period of experimental exposure, mononuclear cells stimulated by LPS, demonstrated increase in the production of cytokines (IL1β, IL2, IL4, IL5, IL6, IL8, IL10, IL12р70, IFN*γ*, TNFα and TNFβ) in compare to the pre-mission values, however the difference was not statistically significant. Nevertheless, his phenomenon indicates a high functional activity of the TLR system of the innate immunity during 520-days confinement (71).

### Experiments With a Confinement in Other Chambers and Facilities

#### 60-Days Confinement, Gender-Mixed Crew, Aged From 26 to 38 Years

In the mid-1990s, the research team from the European Astronaut Centre (EAC) of the European Space Agency in Cologne conducted the experiments on 60-days isolation of a gender-mixed crew of 9 test-volunteers in a sealed facility specially designed on the basis of 2 diving pressure chambers. They studied a rather wide range of parameters characterizing the dynamics of immunological post-isolation changes: the number of CD2^+^, CD3^+^, CD4^+^, CD8^+^, CD19^+^, and CD56^+^ cells, the proliferation of mitogen-induced T cells, the concentration of IFNα and IFNγ in the blood plasma, and a number of bactericidal proteins of plasma and the complement system. The researchers have not identified any significant differences of all the measured parameters compared to baseline (75).

#### 10-Days Confinement, Male Crew, Aged From 20 to 27 Years

In 2004 in Japan, another study of the short-term confinement effects on the immune system was conducted in a sealed facility based on a diving chamber. The experiment involved 5 test-volunteers.

##### Lymphocytes

A tendency to an increase in the proportion of CD69^+^ NK cells by the end of the experiment was observed.

##### Monocytes and Granulocytes

The relative quantity of granulocytes and monocytes increased at the beginning of the confinement, and the number of granulocytes continued increasing throughout the experiment, while the rise of monocytes stabilized. The proportion of monocytes gradually decreased during the confinement. The absolute quantity of all leukocytes also increased during the experiment, although not significantly. Changes in the parameters of innate immunity correlated well with an increase in the level of anxiety calculated on the basis of psychological tests, which indicates these changes as a marker of the immune system’s response to stress. This study clearly demonstrates the need to assess the psychological state of the subjects during the period of experimental confinement in order to identify the degree and nature of the impact of psychological factors on the functioning of the immune system (76).

#### 14-Days Confinement, Male Crew, Aged From 29 to 41 Years

One more project aimed at the studying of the effects of confinement conditions on the human body is the NASA Extreme Environment Mission Operations (NEEMO) project proposed by NASA specialists. In this project participated six male test subjects. It is a submarine module Aquarius, previously used as an apparatus for studying the depths of the sea and the bottom of the oceans, which was later converted into a habitable living module located off the coast of Florida at a depth of 20 meters. This setup was mainly used to study the effects of short-term (up to 14 days) confinement simulating the flights on a Space Shuttle-class of spacecraft.

##### Leucocytes

The absolute leukocyte count showed an increase during experiment but without statistical significance.

##### Monocytes and Granulocytes

Monocytes and granulocytes were increased (significant for monocytes on the 13^th^ day of the experiment). The expression of the β2-Integrin CD11b on granulocytes significantly increased at the 7^th^ and 13^th^ days of exposure as simultaneously the L-selectin CD62L showed a significant shedding.

##### Lymphocytes

Lymphocyte count was significantly decreased on the 7^th^ day of the experiment. At the same time an increase in the relative quantity of cells in the pool of memory T cells were found. The researchers did not reveal any shifts in the CD4/CD8 populations ratio.

##### Cytokines

During the confinement periods (starting from 7-10 days), more than half of the crew members demonstrated a significant decrease in the production of cytokines by lymphocytic cells. However, at the final stages of the experiment and in the post-isolation period, the dynamics of the expression of such cytokines as IFNγ, IL-5, IL-10, IL-2, and IL-6 by the pool of lymphocytes was inverted, as their production increased significantly compared to baseline. In addition, during the experiment, the production of cytokines by monocytes (IL-6, TNFα, IL-10, IL-8, IL-1b) increased.

Also, more than 40% of the crew had an increased titer of antibodies to the Epstein-Barr virus. Since the observed changes are in many respects similar to those observed in real space flight, this model is considered by researchers to be a successful ground-based analogue simulating the impact of stress factors in space flight (77, 78).

It is worth to discuss residence at the polar station in Antarctica as a special type of a model for long-term isolation in extreme conditions. Its uniqueness lies in the fact that people started to live there only at the beginning of the twentieth century, and there is still no permanent population in Antarctica. Today, despite the advanced modern technologies, transport, construction, communications, etc., the hostile, dangerous and unfamiliar environment of Antarctica with its complete physical isolation, cold and pronounced photoperiodicity (i.e., the presence of long periods of round-the-clock illumination or darkness), is possibly the most extreme and by far the most isolated area on Earth (3). Many of the psychosocial stressors encountered by expedition members during wintering in Antarctica are very similar in nature and degree of impact to those faced by astronauts during a long-term space flight (3, 51). These factors include, first of all, a high degree of isolation of the entire group of researchers from the rest of the world, social and physical constraint, sensory monotony, shift of circadian rhythms during the polar day or night, limited telecommunications, psychological stress associated with minimal rescue capabilities in case of unforeseen threats to health and life in the extremely unfavorable environmental conditions (3). This dependence on technology and external supplies for survival in Antarctica is in essence similar to the dependence of astronauts on advanced technologies for food supply, living space, environmental control, communication (with family, professional colleagues). Despite the inability to simulate the microgravity conditions of deep space or the reduced gravity of other planets (the Moon or Mars), as well as a number of other space factors, such as radiation, Antarctica, nevertheless, is an excellent environment for reproducing an analogue of total isolation, characteristic of space missions. That is why the broadest possible study of the psychological and physiological changes occurring in the human body during Antarctic expeditions, including adaptive changes in the immune system, is currently a highly relevant issue of advanced scientific research in the context of preparing mankind for long-term space missions.

#### 365-Days Confinement, Gender-Mixed Crew, Aged From 24-44 (Females); 26-55 (Males)

During the one-year stay of the gender-mixed expedition (10 females and 16 males) at the German polar station Neumayer III, a number of changes in the immunological reactions of its participants were noted, the most striking changes were noted during the polar winter. Interestingly, no sex-depended differences during the experiment were observed (51). Baseline data collection (BDC) was performed in October.

##### Leucocytes

Significant increase in total level of leucocytes in compare to BDC was observed from February to May and from July to October.

##### Granulocytes

Significant increase to baseline for granulocytes were found in males throughout the whole deployment for males and for females from June to November, except of October.

##### Lymphocytes

Lymphocytes were significantly increased in males from April to November and for females from June to November.

##### Cytokines

Basal release of the cytokines (INF-γ, IL-10, IL-2, and TNF) demonstrated a significant increase to baseline only for IL-2 in females in April. Nevertheless, all cytokines showed an increasing trend through the whole experiment. After stimulation by fungal antigens significant difference (increased concentration) was revealed for INF-γ from June to August for male participants. No significant changes during experiment were observed for IL-2 and TNF. INF-γ significantly increased in males and females after the experiment. Cytokine profile after stimulation with the mitogen PWM revealed significant difference for INF-γ in females in April and June. IL-10 was increased in all participants, however significant difference was observed in females in April. For IL-2 and TNF no significant differences were shown (51).

Another study was carried out not only under the conditions of Antarctica, but also under the conditions of the alpine station Concordia (3,233 m above sea level), where in addition to other extreme factors residents suffer from hypobaria (79).

#### 365-Days Experiment, Male Participants, Age Not Presented

During the Antarctic winter-over of fourteen crewmembers, changes in immune systems were observed.

##### Leucocytes

No significant changes were shown during the expedition.

##### Monocytes and Granulocytes

Significantly increased level of absolute count of granulocytes and monocytes on the 4^th^ and 7^th^ months of the experiment was revealed.

##### Lymphocytes

The total level of lymphocytes was increased after one month, on the 4^th^, 8^th^ months of expedition. The increase in the number of lymphocytes was determined mainly by the fraction of B cells, while the relative content of T cells gradually decreased, a particularly clear drop in the number of cells was noted or naïve T cells fraction. In the summer period, an increase in the proportion of mitogen-activated CD69^+^ and HLA-DR^+^ T cells in the 24h culture was observed.

##### Cytokines

During the entire expedition, a decrease in the level of GM-CSF and IL-1α cytokines was noted. In winter, the levels of TNFα and IL-10 increased upon their activation with LPS in whole blood culture; when activated with a mixture of fungal and bacterial antigens, an increase in IFNγ, TNFα, IL-2 in the cultures was noted. The most powerful oxidative activity of granulocytes was also observed in winter. At the beginning of summer, there was a short-term release of IL-1b in activated whole blood cultures, gradually decreasing by the end of summer. When lymphocytes were cultured with the addition of PWM (pokeweed mitogen), an increase in anti-inflammatory IL-10 was observed. By the end of the expedition, a decrease in the expression of certain genes associated with adhesion, cell-cell interactions *via* adaptor molecules, and signal transduction cascades of immunocompetent cells was observed (79).

During one more expedition to Concordia in summer blood, saliva, and urine samples were collected from the 9 members of the expedition, aged from 26 to 58 years. There was a minor increase in the relative quantity of granulocytes in the beginning of the expedition (in the first 7 days), then their amount gradually decreased. During the whole expedition the oxidative activity of granulocytes increased when they were incubated with a specific activator. Also, during the first week of the expedition an increase in the quantity of CD26L+ granulocytes was observed in the conditions of incubation (80).

Similar studies are not possible in the Arctic region, where there is a lack of isolated research stations staffed year-round. However, there are ‘summer deployment’ Arctic stations with staffing of shorter duration, with environmental characteristics more akin to planetary surface exploration Pilot studies have revealed some immune dysregulation does occur associated with Arctic deployment which may create a utility for Arctic/Antarctic deployment similar to short/long duration spaceflight (81). A brief list of described isolation experiments is shown in **Table 2**.

**Table 2:** List of isolation Experiments.

Author	Experiment	Duration	Explored parameters	Key findings
(53)	Moon-2015	9 days	Absolute and relative lymphocyte count in peripheral blood, absolute and relative granulocyte count in peripheral blood; percentage and absolute CD3^+^, CD3^+^ CD4^+^, CD3^+^CD8^+^, CD19^+^, CD3^-^CD16^+^, CD56^+^ lymphocyte count total leukocyte count, absolute and relative monocyte and granulocyte counts in peripheral blood; percentage and absolute count of TLR2^+^, TLR4^+^, TLR6^+^-monocytes and granulocytes	Different changes in absolute and relative cells of innate and adaptive immunity were observed during the experiment.
(52)	SIRIUS-17	17 days	Total content of leukocytes, absolute and relative monocyte and granulocyte count in peripheral blood; percentage and absolute number of TLR1^+^, TLR2^+^, TLR3^+^, TLR4^+^, TLR5^+^, TLR6^+^, TLR8^+^, TLR9^+^-monocytes and granulocytes; level of CD14^-^/CD16^-^,CD123 ^+^, CD85k ^+^ plasmacytoid DCs; content of mature CD86 ^+^ CD83 ^+^ CD14^−^ DCs.	Gender independent dramatic decrease in the relative quantity of TLR4^+^, TLR5^+^, TLR6^+^ monocytes, especially, TLR3^+^, TLR8^+^, TLR9^+^ cells, by the time of the finishing of the experiment compared to baseline values.
(63)	HUBES	135 days	Phytohemagglutinin reactivity of T-lymphocytes; suppressor activity of T-lymphocytes, induced by concanavalin A; functional activity of T-helper lymphocytes, evaluated by the xenogeneic graft-versus-host reaction; сytotoxicity of NK cells; mitogen-induced production of interleukin-2, a-interferon, γ-interferon, and osteoclast activating factor; levels of immunoglobulins (A, G, M) in blood serum; levels of rheumatoid factor and DNA autoantibody titers; sensitization to bacterial and chemical allergens identified by the capacity of cells to produce a humoral mediator, leukocyte migration inhibition factor (LIF).	Decrease in the level of A, M, and G immunoglobulins in the blood serum, a decrease in the functional activity of T-lymphocytes, suppression of the cytotoxic activity of natural killers, signs of sensitization to bacterial allergens and formaldehyde during the isolation.
(64)	SFINCSS-99	110 and 240 days	Functional state of T cells, assessed using the reaction of blast transformation under the action of PHA; number of immunoglobulins (IgG, IgA, and IgM)	Functional activity of T-cells was reduced on average starting from the 4th month of the confinement. Decreased level of immunoglobulins (IgM, IgG) during the experiment.
(1)	SFINCSS-99	110 and 240 days	Level of CD3^+^CD4^+^, CD3^+^CD8^+^, CD19^+^, CD16^+^CD56^+^- lymphocytes; level of plasma cytokines (IL6, IFNγ); delayed type hypersensitivity reaction of the skin against a panel of standardized recall antigens. Concentration of polymorphonuclear leukocytes (PMNL) and monocytes; CD18/CD11b expression level on PMNL; superoxide anion production by phagocytes in diluted whole blood.	The ratio of T-helper to T-suppressor cells during the mission decreased; enhanced expression of beta2-integrins by circulating granulocytes during the experiment.
(65)	«Mars-500»	105 days	Content of lymphocytes in peripheral blood; absolute and relative content of CD3^+^, CD4^+^, CD8^+^, CD19^+^, CD25^+^, CD45RA^+^, CD45RO^+^, HLA-DR^+^- lymphocytes; content of CD69^+^, CD25^+^- lymphocytes in PHA-stimulated cultures of mononuclear cells; levels of IFNγ, IL2, IL10, IL6, IL4, IL5 and TNF in supernatant of PHA-stimulated cell cultures; levels of IFNγ, IL2, IL10, IL6, IL4, IL5, and TNF in plasma; amount of immunoglobulins (IgG, IgA, and IgM) in plasma; content of monocytes and granulocytes in peripheral blood levels of IFNγ, IL2, IL10, IL6, IL4, IL5, and TNF in plasma.	Increased absolute and relative number of CD4+CD25^Bright^ lymphocytes on the 7^th^ and 105^th^ days of isolation, decreased B-cells on the 16th and 70th days of the experiment, increased Il-8 level during the mission.
(70)	«Mars-500»	105 days	Concentrations of lymphocytes; concentrations of IL-1b, IL-2, IL-4, IL-5, IL-6, IL-7, IL-8, IL-10, IL-12¸ IL-13, G-CSF, GM-CSF, IFNγ, MCP-1, MIP-1b, TNFα, TGFβ in plasma; concentrations of polymorphonuclear leukocytes; hydrogen peroxide (H_2_O_2_) production by polymorphonuclear leukocytes in stimulated cell cultures; adhesive and phagocytic capabilities of polymorphonuclear leukocytes; level of CD62L+- polymorphonuclear leukocytes; concentrations of IL-1b, IL-2, IL-4, IL-5, IL-6, IL-7, IL-8, IL-10, IL-12¸ IL-13, G-CSF, GM-CSF, IFNγ, MCP-1, MIP-1b, TNFα, TGFβ in plasma.	Suppression of the phagocytic function of mononuclear cells during the experiment.
(71)	«Mars-500»	520 days	Number of lymphocytes; level of NK-cells; level of CD69+-NK-cells in stimulated cell cultures; level of CD19 ^+^, CD3 ^+^, CD3 ^+^ CD4 ^+^, CD3 ^+^ CD8 ^+^, CD3 ^+^ CD25^+^, CD4 ^+^ CD25 ^+ bright^, CD4 ^+^ CD45RA^+^, CD4 ^+^ CD45RO ^+^ lymphocytes; level of CD19^+^CD69^+^, CD3^+^CD69^+^, CD3^+^CD25^+^- lymphocytes in stimulated cell cultures; serum immunoglobulin level (IgA, IgM, IgG); serum and stimulated cells cultures cytokine level (IL-1α, IL-1β, IL-2, IL-4, IL-5, IL-6, IL-8, IL-10, IL-12p70, IFN-γ, TNF α and TNF β). Number of leukocytes, monocytes and granulocytes; amount of TLR2^+^, TLR 4^+^, TLR6^+^, CD36^+^, CD206^+^, CD24^+^, CD54^+^, CD16^+^- monocytes and granulocytes; level of HSP-70 in serum; cytokine level (IL-1α, IL-1β, IL-2, IL-4, IL-5, IL-6, IL-8, IL-10, IL-12p70, IFN-γ, TNF α and TNF β) in serum and in stimulated cells cultures.	Wave-like changes in the vast majority of the studied parameters that characterize the state of innate and adaptive immunity.
(72)	«Mars-500»	520 days	Absolute white blood cell count and the percentage of each type of white blood cells; level of CD19 +, CD3 +, CD3 ^+^ CD4 ^+^, CD3 ^+^ CD8 ^+^, CD3^-^CD16^+^CD56^+^- lymphocytes; level of IL-2, IL-6, IFN-γ, TNFα in cells cultures stimulated by Epstein-Barr-Virus (EBV)-lysate; amount of antibodies to EBV. Absolute white blood cell count and the percentage of each type of white blood cell; level of IL-2, IL-6, IFN-γ, TNF α in cell cultures stimulated by Epstein-Barr-Virus (EBV)-lysate;	An increase in cytokine production by immunocompetent cells was observed during the mission upon their activation by the Epstein-Barr viral particles. The concentration of IFNγ and TNF were increased as well.
(75)		60 days	Count of CD2^+^, CD3^+^CD4^+^, CD3^+^CD8^+^, CD19^+^, CD56^+^, CD45RA^+^, CD45RO^+^, CD25^+^-cells; proliferation assay of lymphocytes in PHA-stimulated cell cultures; level of CD3^+^CD25^+^-cells in stimulated cultures; NK-cell activity; level of IFN-γ, IFN-α in stimulated cells cultures.	No significant changes in explored parameters.
(76)		10 days	lymphocyte percentage; level of NK-cells and CD69^+^-NK-cells; total leukocyte counts, granulocyte and monocyte percentage.	Increased relative quantity of granulocytes and monocytes at the beginning of the confinement, and the number of granulocytes continued increasing throughout the experiment, while the rise of monocytes stabilized.
(77, 78)	NEEMO	14 days	lymphocyte percentage; count of CD3^+^CD4^+^, CD3^+^CD8^+^-cells; production of IFNγ, IL-5, IL-10, IL-2, IL-6, TNFα by lymphocytes; amount of antibodies to EBV total leukocyte counts, granulocyte and monocyte percentage; production of IL-6, TNFα, IL-10, IL-8, IL-1b by monocytes.	Monocytes and granulocytes were increased (significant for monocytes on the 13th day of the experiment). The expression of the β2-Integrin CD11b on granulocytes significantly increased at the 7th and 13th days of exposure as simultaneously the L-selectin CD62L showed a significant shedding.
				Lymphocyte count was significantly decreased on the 7th day of the experiment. At the same time an increase in the relative quantity of cells in the pool of memory T cells were found. Cytokines decreased starting from one week of the experiment.
(51)	Antarctic expedition in Neumayer III station.	1 year	lymphocyte percentage; level of IFN-γ, IL-10, IL-2, and TNF in stimulated cells cultures; total leukocyte counts, granulocyte percentage.	No differences in immunological reaction between male and female participants were observed. All measured immunological parameters revealed a tendency to increase.
(79)	Antarctic expedition in Concordia station.	1 year	Lymphocyte counts and percentage; count of CD3^+^CD4^+^, CD3^+^CD8^+^, CD19^+^, NK-cells, CD4 and CD8 naïve, bulk memory, central memory-cells; level of CD4^+^CD69^+^, CD8^+^CD69^+^, CD4^+^CD25^+^, CD8^+^CD25^+^, CD4^+^HLA-DR^+^, CD8^+^HLA-DR^+^- lymphocytes in stimulated cells cultures; level of G-CSF, CXCL/ENA-78, IL-1ra, in plasma; level of cytokines in stimulated cells cultures; CD272, CD150, CD28, CD80 gene expression; total leukocyte counts, granulocyte counts and percentage; level of G-CSF, CXCL/ENA-78, IL-1ra in plasma; level of IFN-γ, IL-10, IL-2, and TNF in stimulated whole blood cultures; evaluation of oxidative burst; CD80 gene expression.	T-cells were significantly decreased during the expedition, the majority of other immune effectors were increased during the experiment.
(80)	Antarctic expedition in Concordia station.	4 month	lymphocyte counts and percentage; total leukocyte counts, granulocyte counts and percentage; evaluation of oxidative burst; level of CD62L expression on granulocytes.	During the whole expedition the oxidative activity of granulocytes increased. Also, during the first week of the expedition an increase in the quantity of CD26L+ granulocytes was observed.

## Conclusion

From the extensive data considered above we can conclude that the changes in immunological reactions and the immune status of the human body observed in model experiments of isolation and confinement are in many respects similar to those occurring under the conditions on board the real spacecraft. This clearly indicates the high validity of using such experimental models for ground-based studies of the influence of space flight factors. This is becoming especially relevant in the light of long-term space missions planning, in which such preliminary detailed modeling will reveal and, with high probability, will allow to prevent a number of serious risks to the health and normal performance of future space explorers. In our opinion, all the drawbacks of such experimental studies, in particular, their high cost, long duration, complexity of processing, analysis and interpretation of the colossal array of data received, are repaid hundredfold by the reduction of risks threatening not only the failure of the research mission or unsatisfactory performance of its main tasks, but also the life and health of astronauts.

The very wide individual variation in the changes of the immunological parameters observed in the isolation and confinement experiments is noteworthy. One of the promising lines of investigation in isolation and confinement models is the search for a “universal set of parameters” or a set of interdependent changes in different parameters, considered as a single functional ensemble. Focusing on these sets will make it possible to produce a fairly simple and accurate express diagnostics of the current state of immunity, and to forecast the development of this state in time. In addition, the researches available to date focus primarily on the changes occurring in the adaptive immunity. In order to develop the above-mentioned approach, it is necessary to conduct experiments of various degrees of duration, with a more thorough investigation of the aspects and features of immunity which are insufficiently studied in the context of space flights and isolation. In this regard, the study of innate immunity, especially, the pattern-recognition receptors and dendritic cells as a functional link between innate and adaptive parts of the immune system, as well as the changes in gene expression of various populations of immunocompetent cells, is of particular interest.

Since the immune system interacts closely with other regulatory systems, such as the nervous and endocrine systems, for the task set above, it is also necessary to conduct the studies aimed at the elucidating of the simultaneous changes in the parameters of these systems in the conditions of isolation and confinement. A thorough correlation analysis of data on changes in all three systems (immune, nervous, and endocrine), on changes in blood biochemical parameters, as well as correlation analysis of data on changes in immunological parameters of various components of immunity itself is required.

As described above, spaceflight stressors (microgravity, isolation, stress, radiation) results in a pattern of immune alterations that can result in increased clinical risks to Cosmonauts or Astronauts participating in deep space missions. The pattern of dysregulation is similar to those associated with some terrestrial disease, including Zoster patients (82). Countermeasures development is required. By definition, human spaceflight studies are severely operationally constrained. Limited sampling opportunities, limited capability to process or analyzed samples on orbit and limits in up/down mass bound biological investigations. Ground analogs of spaceflight, in addition to affording the opportunity to greatly expand scientific analysis and further characterize physiological dysregulation, also represent an opportunity to triage various spaceflight countermeasures without the constraints of an actual spaceflight experiment (34). Chamber isolation in particular, given that the analog is typically nearby to full laboratory or medical facilities, allows an almost unconstrained scientific approach as contrasted with Antarctica winter over or undersea deployment. The countermeasure options include SAHC, various training physical loads, use of stress relieving exercises, and taking of pharmacological medicines, probiotics or vitamins (2). It is necessary to evaluate both the principled appropriateness of the countermeasures in the conditions of experimental confinement, and the schedule of their application, as well as efficient doses, combinations and duration of application. Moreover, all this should be done with regard to the wide individual variations of reactions of crew members and to the fundamental data obtained from a thorough investigations of the above-mentioned components of the immune system. There is some evidence that countermeasures already deployed to ISS have benefited immunity (35). However, given the increased magnitude of both stressors and clinic risk associated with deep space missions, and that most ISS countermeasures do not translate to deep space vehicles, a novel international immune countermeasure protocol has been developed by an international team of translational space scientists (83). Prolonged closed chamber confinement in a high-fidelity vehicle simulation such as NEK/SIRIUS may be an ideal platform for such a countermeasure validation.

Execution of terrestrial confinement studies will make it possible to obtain not only a unique array of the latest fundamental scientific data, but also to develop countermeasures and practical training systems for astronauts, especially for those who will join the upcoming interplanetary expeditions and inhabit planetary and near-planetary stations. Moreover, in case of the successful testing of such a system in the most extreme conditions of the outer space it can be well adapted for the needs of terrestrial medicine in extreme situations, support and rehabilitation of people in conditions of forced isolation and hypokinesia, including the commercially based.

## Author Contributions

All authors listed have made a substantial, direct, and intellectual contribution to the work, and approved it for publication.

## Funding

The study was supported by the Ministry of Science and Higher Education of the Russian Federation under the agreement № _075-1502020-919 from 16.11.2020 about the grant in the form of subsidy from the federal budget to provide government support for the creation and development of a worldclass research center “Pavlov Center for Integrative Physiology to Medicine, High-tech Healthcare and Stress Tolerance Technologies”.

## Conflict of Interest

The authors declare that the research was conducted in the absence of any commercial or financial relationships that could be construed as a potential conflict of interest.

## References

[1] Choukèr A Smith L Christ F Larina I Nichiporuk I Baranov V. Effects of Confinement (110 and 240 Days) on Neuroendocrine Stress Response and Changes of Immune Cells in Men. J Appl Physiol (1985) (2002) 92(4):1619–27. 10.1152/japplphysiol.00732.2001 11896029

[2] Crucian B Chouker A Simpson R Mehta S Marshall G Smith S. Immune System Dysregulation During Spaceflight: Suggested Countermeasures for Deep Space Missions. Front Immunol (2018) 9:1437. 10.3389/fimmu.2018.01437 30018614 PMC6038331

[3] Salam A. Exploration Class Missions on Earth: Lessons Learnt From Life in Extreme Antarctic Isolation and Confinement. Stress Challenges Immun Space (2020) 693–707. 10.1007/978-3-030-16996-1_38

[4] Konstantinova IV. The Immune System in Extreme Conditions. Space Immunol (1988) 289. 3254527

[5] Rykova MP Antropova EN Meshkov DO. Results of Immunological Examination of Cosmonauts During the Period of Readaptation After Space Flight. Orbital Station MIR (2001) 1:615.

[6] Konstantinova IV Rykova MP Lesnyak AT Antropova EA. Immune Changes During Long-Duration Missions. J Leukoc Biol (1993) 54(3):189–201. 10.1002/jlb.54.3.189 8371048

[7] Rykova M Meshkov D Antropova E Polykov V Schaffar L Schmitt D. The Effects of Microgravity on the Immune System. Int. Symp. In: International Scientific Cooperation Onboard MIR Proceedings (2001). p. 237.

[8] Meehan R Whitson P Sams C. The Role of Psychoneuroendocrine Factors on Spaceflight-Induced Immunological Alterations. J Leukoc Biol (1993) 54(3):236–44. 10.1002/jlb.54.3.236 8371053

[9] Stowe RP Sams CF Mehta SK Kaur I Jones ML Feeback DL. Leukocyte Subsets and Neutrophil Function After Short-Term Spaceflight. J Leukoc Biol (1999) 65(2):179–86. 10.1002/jlb.65.2.179 10088600

[10] Cogoli A. The Effect of Space Flight on Human Cellular Immunity. Environ Med (1993) 37(2):107–16. 12211252

[11] Gmünder FK Konstantinova I Cogoli А Lesnyak А Bogomolow W Grachov AW. Cellular Immunity in Cosmonauts During Long Duration Spaceflight on Board the Orbital MIR Station. Aviat Space Environ Med (1994) 65(5):419–23. 8024524

[12] Taylor GR Janney RP. *In Vivo* Testing Confirms a Blunting of the Human Cell-Mediated Immune Mechanism During Space Flight. J Leukoc Biol (1992) 51(2):129–32. 10.1002/jlb.51.2.129 1431548

[13] Mehta SK Laudenslager ML Stowe RP Crucian BE Sams CF Pierson DL. Multiple Latent Viruses Reactivate in Astronauts During Space Shuttle Missions. Brain Behav Immun (2014) 41:210–7. 10.1016/j.bbi.2014.05.014 24886968

[14] Mehta SK Pierson DL. Reactivation of Latent Herpesviruses in Cosmonauts During Soyuz Taxi Mission. Micrograv Sci Tech (2007) XIX:215. 10.1007/BF02919485

[15] Crucian B Stowe R Mehta S Uchakin P Quiriarte H Pierson D. Immune System Dysregulation Occurs During Short Duration Spaceflight on Board the Space Shuttle. J Clin Immunol (2013) 33(2):456–65. 10.1007/s10875-012-9824-7 23100144

[16] Crucian BE Zwart SR Mehta S Uchakin P Quiriarte HD Pierson D. Plasma Cytokine Concentrations Indicate That *In Vivo* Hormonal Regulation of Immunity Is Altered During Long-Duration Spaceflight. J Interferon Cytokine Res (2014) 34(10):778–86. 10.1089/jir.2013.0129 PMC4186776 24702175

[17] Manié S Konstantinova I Breittmayer JP Ferrua B Schaffar L. Effects of Long Duration Spaceflight on Human T Lymphocyte and Monocyte Activity. Aviat Space Environ Med (1991) 62(12):1153–8. 1755796

[18] Buravkova LB Rykova MP Grigorieva V Antropova EN. Cell Interactions in Microgravity: Cytotoxic Effects of Natural Killer Cells In Vitro. J Gravit Physiol (2004) 11(2):177–80. 16237828

[19] Konstantinova IV Rykova M Meshkov D Peres C Husson D Schmitt DA. Natural Killer Cells After ALTAIR Mission. Acta Astronaut (1995) 36(8-12):713–8. 10.1016/0094-5765(95)00161-1 11541007

[20] Meshkov D Rykova M. The Natural Cytotoxicity in Cosmonauts on Board Space Stations. Acta Astronaut (1995) 36(8-12):719–26. 10.1016/0094-5765(95)00162-X 11541008

[21] Rykova MP Sonnenfeld G Lesnyak AT Taylor GR Meshkov DO Mandel AD. Effect of Spaceflight on Natural Killer Cell Activity. J Appl Physiol (1985) 73(2):196S–200S. 10.1152/jappl.1992.73.2.S196 1526952

[22] Crucian BE Stowe RP Pierson DL Sams CF. Immune System Dysregulation Following Short- vs Long-Duration Spaceflight. Aviat Space Environ Med (2008) 79(9):835–43. 10.3357/ASEM.2276.2008 18785351

[23] Morukov B Rykova M Antropova E Berendeeva T Ponomaryov S Larina I. T Cell Immunity and Cytokine Production in Cosmonauts After Long-Duration Spaceflights. Acta Astronaut (2011) 68(7-8):739–46. 10.1016/j.actaastro.2010.08.036

[24] Rykova M Antropova E Larina I Morukov B. Humoral and Cellular Immunity in Cosmonauts After the ISS Missions. Acta Astronaut (2008) 63:697–705. 10.1016/j.actaastro.2008.03.016

[25] Cohrs RJ Mehta SK Schmid DS Gilden DH Pierson DL. Asymptomatic Reactivation and Shed of Infectious Varicella Zoster Virus in Astronauts. J Med Virol (2008) 80(6):1116–22. 10.1002/jmv.21173 PMC2938738 18428120

[26] Mehta SK Crucian BE Stowe RP Simpson RJ Ott CM Sams CF. Reactivation of Latent Viruses Is Associated With Increased Plasma Cytokines in Astronauts. Cytokine (2013) 61(1):205–9. 10.1016/j.cyto.2012.09.019 23107825

[27] Stowe RP Kozlova EV Sams CF Pierson DL. Walling DM. Latent and Lytic Epstein-Barr Virus Gene Expression in the Peripheral Blood of Astronauts. J Med Virol (2011) 83(6):1071–7. 10.1002/jmv.22079 21503923

[28] Stowe RP Sams CF Pierson DL. Adrenocortical and Immune Responses Following Short- and Long-Duration Spaceflight. Aviat Space Environ Med (2011) 82(6):627–34. 10.3357/ASEM.2980.2011 21702314

[29] Crucian B Stowe R Quiriarte H Pierson D Sams C. Monocyte Phenotype and Cytokine Production Profiles Are Dysregulated by Short-Duration Spaceflight. Aviat Space Environ Med (2011) 82(9):857–62. 10.3357/ASEM.3047.2011 21888268

[30] Ponomarev SA Berendeeva TA Kalinin SA Muranova AV. Status of the System of Signaling Pattern Recognition Receptors of Monocytes and Granulocytes in Cosmonauts’ Peripheral Blood Before and After Long-Duration Missions to the International Space Station. Aviakosmicheskaya i Ekologicheskaya Meditsina (Russia) (2016) 50(5):18–23. 10.1134/S0362119717070167 29553590

[31] Crucian B Stowe RP Mehta S Quiriarte H Pierson D Sams C. Alterations in Adaptive Immunity Persist During Long-Duration Spaceflight. NPJ Microgravity (2015) 3(1):15013. 10.1038/npjmgrav.2015.13 PMC5515498 28725716

[32] Mehta SK Laudenslager ML Stowe RP Crucian BE Feiveson AH Sams CF. Latent Virus Reactivation in Astronauts on the International Space Station. NPJ Microgravity (2017) 12(3):11. 10.1038/s41526-017-0015-y PMC5445581 28649633

[33] Makedonas G Chouker A Mehta S Simpson R Stowe R Sams C. Mechanistic Clues to Overcome Spaceflight-Induced Immune Dysregulation. Curr Pathobiol Rep (2018) 6:185–92. 10.1007/s40139-018-0178-6

[34] Crucian B Simpson RJ Mehta S Stowe R Chouker A Hwang SA. Terrestrial Stress Analogs for Spaceflight Associated Immune System Dysregulation. Brain Behav Immun (2014) 39:23–32. 10.1016/j.bbi.2014.01.011 24462949

[35] Crucian BE Makedonas G Sams CF Pierson DL Simpson R Stowe RP. Countermeasures-Based Improvements in Stress, Immune System Dysregulation and Latent Herpesvirus Reactivation Onboard the International Space Station - Relevance for Deep Space Missions and Terrestrial Medicine. Neurosci Biobehav Rev (2020) 115:68–76. 10.1016/j.neubiorev.2020.05.007 32464118

[36] Grigoriev AI Morukov BV. Mars Is Getting Closer. Sci Russia (2011) 1:4.

[37] Belkaid Y Hand TW. Role of the Microbiota in Immunity and Inflammation. Cell (2014) 157:121–41. 10.1016/j.cell.2014.03.011 PMC4056765 24679531

[38] Wu H-J Wu E. The Role of Gut Microbiota in Immune Homeostasis and Autoimmunity. Gut Microbes (2012) 3:4–14. 10.4161/gmic.19320 22356853 PMC3337124

[39] Schirmer M Smeekens SP Vlamakis H Jaeger M Oosting M Franzosa EA. Linking the Human Gut Microbiome to Inflammatory Cytokine Production Capacity. Cell (2016) 167:1125–36.e8. 10.1016/j.cell.2016.10.020 27814509 PMC5131922

[40] Daïen CI Pinget GV Tan JK Macia L. Detrimental Impact of Microbiota Accessible Carbohydrate-Deprived Diet on Gut and Immune Homeostasis: An Overview. Front Immunol (2017) 8:548. 10.3389/fimmu.2017.00548 28553291 PMC5427073

[41] Siddiqui R Akbar N Khan NA. Gut Microbiome and Human Health Under the Space Environment. J Appl Microbiol (2021) 130(1):14–24. 10.1111/jam.14789 32692438

[42] Ilyin VK Kiryukhina NV. Disruption of the Colonization Resistance Syndrome in Humans in Altered Habitats and its Prevention. Acta Naturae (2014) 6:10–8. 10.32607/20758251-2014-6-2-10-18 PMC4115221 25093106

[43] Voorhies AA Mark Ott C Mehta S Pierson DL Crucian BE Feiveson A. Study of the Impact of Long-Duration Space Missions at the International Space Station on the Astronaut Microbiome. Sci Rep (2019) 9(1):9911. 10.1038/s41598-019-46303-8 31289321 PMC6616552

[44] Spencer MJ Cherry JD Powell KR Mickey MR Terasaki PI Marcy SM. Antibody Responses Following Rubella Immunization Analyzed by HLA and ABO Types. Immunogenetics (1977) 4:365–72. 10.1007/BF01575674

[45] Goble FC Konopka EA. Sex as a Factor in Infectious Diseases. Trans NY Acad Sci (1973) 35:32. 10.1111/j.2164-0947.1973.tb01971.x

[46] Klein SL Flanagan KL. Sex Differences in Immune Responses. Nat Rev Immunol (2016) 16:626–38. 10.1038/nri.2016.90 27546235

[47] Hertz D Schneider B. Sex Differences in Tuberculosis. Semin Immunopathol (2019) 41:225–37. 10.1007/s00281-018-0725-6 30361803

[48] Cook MB McGlynn KA Devesa SS Freedman ND Anderson WF. Sex Disparities in Cancer Mortality and Survival. Cancer Epidemiol Biomark Prev (2011) 20(8):1629–37. 10.1158/1055-9965.EPI-11-0246 PMC3153584 21750167

[49] Whitacre CC. Sex Differences in Autoimmune Disease. Nat Immunol (2001) 2:777–80. 10.1038/ni0901-777 11526384

[50] Kennedy AR Crucian BE Huff JL. Effects of Sex and Gender on Adaptation to Space: Immune System. J Women’s Health (2014) 23(11):956–8. 10.1089/jwh.2014.4913 PMC4236064 25401940

[51] Strewe C Moser D Buchheim JI Gunga HC Stahn A Crucian BE. Sex Differences in Stress and Immune Responses During Confinement in Antarctica. Biol Sex Differ (2019) 10(1):20. 10.1186/s13293-019-0231-0 30992051 PMC6469129

[52] Ponomarev S Kutko O Rykova M Kalinin S Antropova E Sadova A. Changes in the Cellular Component of the Human Innate Immunity System in Short-Term Isolation. Acta Astronautica (2020) 166:89–92. 10.1016/j.actaastro.2019.10.012

[53] Ponomarev SA Muranova AV Kalinin SA Antropova EN Rykova MP Koloteva MI. Cell Immunity Indices in Crew Members of the “Moon-2015” Project. Aviakosmicheskaya i Ekologicheskaya Meditsina (2017) 51(2):13–9. 10.1134/S0362119718070125

[54] Grigoriev AI Bugrov SA Bogomolov VV Egorov AD Polyakov VV Tarasov IK. Review of the Main Medical Results of the Annual Flight to the Mir Station. Cosm Biol Aerospace Med (1990) 24(5):3–10. 10.1016/0094-5765(93)90073-6

[55] Kozlovskaya IB Grigoriev AI. Russian System of Countermeasures on Board of the International Space Station (ISS): The First Results. Acta Astronaut (2004) 55(3–9):233–7. 10.1016/j.actaastro.2004.05.049 15806738

[56] Vil-Viliams IF. Principle Approaches to Selection of the Short-Arm Centrifuge Regimens for Extended Space Flight. Acta Astronaut (1994) 33(C):221–9. 10.1016/0094-5765(94)90129-5 11539526

[57] Ilyin VK Novikova ND. Human Microflora Under Conditions of Application of Enterococcus Faecium-Based Autoprobiotics in 105-Day Isolation. Aviakosmicheskaya i Ekologicheskaya Meditsina (2010) 44(4):52–7.

[58] Tartey S Takeuchi O. Pathogen Recognition and Toll-Like Receptor Targeted Therapeutics in Innate Immune Cells. Int Rev Immunol (2017) 36(2):57–73. 10.1080/08830185.2016.1261318 28060562

[59] Lebedev KA Ponyakina ID. Immunology of the Image Recognition Receptors: Integral Immunology. Moscow: Librikom Book House (2009), 256.

[60] Takeda K Akira S. Toll-Like Receptors. Curr Protoc Immunol (2015) 109:14.12.1–14.12.10. 10.1002/0471142735.im1412s109 25845562

[61] Kovalchuk LV Khoreva MV Varivoda AS Pashchenko OE Gracheva LA Bykova LP. Effect of Toll-Like Receptors (TLR) Ligands on In Vitro Synthesis of Proinflammatory Cytokines by Peripheral Blood Mononuclear Cells From Healthy Men and From Patients With Common Variable Immunodeficiency. Zh Mikrobiol Epidemiol Immunobiol (2007) 1:38–42. 17523427

[62] Karnell JL Wu Y Mittereder N Smith MA Gunsior M Yan L. Depleting Plasmacytoid Dendritic Cells Reduces Local Type I Interferon Responses and Disease Activity in Patients With Cutaneous Lupus. Sci Transl Med (2021) 13(595):eabf8442. 10.1126/scitranslmed.abf8442 34039741

[63] Konstantinova IV Antropova EN Meshkov DO Rykova MP Lesnyak A. Immune Resistance of Human During Extended Isolation. Aviakosmicheskaya i Ekologicheskaya Meditsina (1997) 31(4):57–60. 9424200

[64] Polikarpov NA Rykova MP Antropova EN Tsetlin VV Nosovskiy AM Meshkov DO. Some Observations of the State of Immunity-Microflora in Crew Members Under the Conditions of the SFINCSS-99 Experiment in Comparison With the Parameters of Heliomagnetic Activity. Model Experiment With Long-Term Isolation: Problems and Achievements. Moscow: Slovo (2001) 480–90.

[65] Morukov BV Rykova MP Antropova EN Berendeeva TA Ponomarev SA. The Human System of Immunity in the Conditions of 105-Day Isolation and Confinement in Artificial Environment. Aviakosmicheskaya i Ekologicheskaya Meditsina (2010) 44(4):39–46. 10.1134/S0362119712070158

[66] Viktorov AN Novikova ND Deshevaya YA Polikarpov NA Poddubko SV Bragina MP. Results of Microbiological Research. Orbital Station MIR Moscow (2001) 1:121–51.

[67] Ilyin VK Volozhin AI Vikha GV. Colonization Resistance of the Organism Under Altered Habitat Conditions. Moscow: Sci (2005), 280.

[68] Novikova N De Boever P Poddubko S Deshevaya E Polikarpov N Rakova N. Survey of the Environmental Biocontamination Aboard the International Space Station. Res Microbiol (2006) 157(1):5–12. 10.1016/j.resmic.2005.07.010 16364606

[69] Sakaguchi S. Regulatory T Cells: History and Perspective. Methods Mol Biol (2011) 707:3–17. 10.1007/978-1-61737-979-6_1 21287325

[70] Strewe C Muckenthaler F Feuerecker M Yi B Rykova M Kaufmann I. Functional Changes in Neutrophils and Psychoneuroendocrine Responses During 105 Days of Confinement. J Appl Physiol (2015) 118(9):1122–7. 10.1152/japplphysiol.00755.2014 25678697

[71] Morukov BV Rykova MP Antropova EN Berendeeva TA Morukov IB Ponomarev SA. Immunological Aspects of a Space Flight to Mars. Hum Physiol (2013) 39(2):126–35. 10.1134/S0362119713020102 23789382

[72] Yi B Rykova M Feuerecker M Jäger B Ladinig C Basner M. 520-D Isolation and Confinement Simulating a Flight to Mars Reveals Heightened Immune Responses and Alterations of Leukocyte Phenotype. Brain Behav Immun (2014) 40:203–10. 10.1016/j.bbi.2014.03.018 24704568

[73] Lebedev KA Ponyakina ID Kozachenko NV. The Concept of the Norm in Assessing the Immune Status of a Person. Hum Physiol (1989) 15(6):34. 2632325

[74] Husson D Abbal M Tafani M Schmitt DA. Neuroendocrine System and Immune Responses After Confinement. Adv Space Biol Med (1996) 5:93–113. 10.1016/S1569-2574(08)60055-6 8814815

[75] Schmitt DA Peres C Sonnenfeld G Tkackzuk J Arquier M Mauco G. Immune Responses in Humans After 60 Days of Confinement. Brain Behav Immun (1995) 9(1):70–7. 10.1006/brbi.1995.1007 7620212

[76] Shimamiya T Terada N Hiejima Y Wakabayashi S Kasai H Mohri M. Effects of 10-Day Confinement on the Immune System and Psychological Aspects in Humans. J Appl Physiol (2004) 97:920–4. 10.1152/japplphysiol.00043.2004 15145927

[77] Strewe C Crucian BE Sams CF Feuerecker B Stowe RP Choukèr A. Hyperbaric Hyperoxia Alters Innate Immune Functional Properties During NASA Extreme Environment Mission Operation (Neemo). Brain Behav Immun (2015) 50:52–7. 10.1016/j.bbi.2015.06.017 26116982

[78] Strewe C Crucian B Mehta S Feuerecker M. (2012). In: Immune Functions during NASA Extreme Environment Mission Operations (NEEMO): the Role of Hyperoxic Stress. Life in Space for Life on Earth – 12th European Life Sciences Symposium 33rd Annual International Gravitational Physiology Meeting. Aberdeen, United Kingdom.

[79] Feuerecker M Crucian BE Quintens R Buchheim JI Salam AP Rybka A. Immune Sensitization During 1 Year in the Antarctic High-Altitude Concordia Environment. Allergy (2019) 74(1):64–77. 10.1111/all.13545 29978486

[80] Feuerecker M Crucian B Salam AP Rybka A Kaufmann I Moreels M. Early Adaption to the Antarctic Environment at Dome C: Consequences on Stress-Sensitive Innate Immune Functions. High Alt Med Biol (2014) 15(3):341–8. 10.1089/ham.2013.1128 25099674

[81] Crucian B Lee P Stowe R Jones J Effenhauser R Widen R. Field Immune Assessment During Simulated Planetary Exploration on Devon Island, High Arctic. BMC Immunol (2007) 8:7. 10.1186/1471-2172-8-7 17521440 PMC1890299

[82] Makedonas G Kunz HE Mehta SK Tyring SK Vangipuram R Quiriarte H. Immune System Dysregulation and Oral Viral Shedding in Zoster Patients: Relevance for Spaceflight. Life Sci Space Res (2020) 25:119–28. 10.1016/j.lssr.2019.10.001 32414485

[83] Makedonas G Mehta S Choukèr A Simpson RJ Marshall G Orange JS. Specific Immunologic Countermeasure Protocol for Deep-Space Exploration Missions. Front Immunol (2019) 10:2407. 10.3389/fimmu.2019.02407 31681296 PMC6797618

